# P-586. 2025 Nipah Outbreaks in Bangladesh: Clinical Patterns, Emerging Risks, and Future Preparedness in an Expanding Epidemiologic Landscape

**DOI:** 10.1093/ofid/ofaf695.800

**Published:** 2026-01-11

**Authors:** Md Mustafizur Rahman, Sharmin Sultana, Pronesh Dutta, Md Sazzad Hossain, Wasik Rahman Aquib, Sumaya Sachi, Kyaw Thowai Prue Prince, Ratna Das, Nafisa Tasnim Oyshee, Asma Jamal Antara, Shadman Sakib Choudhury, Anika Farzin, Mohammad Rezaul Karim, A K M Dawlat Khan, Tonmoy Sarkar, Nabila Nujhat Chowdhury, Md Arif Khan, Fariha Masfiqua Malek, Umme Fatema, Hosneara Parvin, Mostafa Nahian Habib, Jahid Hasan, Nurun Nahar Chisty, Muhammad Rashedul Alam, Mohammad Ariful Islam, Nisharggo Niloy, Shah Jawad Bin Mahmood, Ayesha Siddika, Md Mahfuzur Rahman, Mintu Chowdhury, Md Omar Qayum, Ariful Islam, Mohammad Enayet Hossain, Christina Spiropoulou, Trevor Shoemaker, Mohammed Ziaur Rahman, Sayera Banu, Lisa Hensley, Syed Moinuddin Satter, Joel M Montgomery, Tahmina Shirin

**Affiliations:** icddr,b (International Centre for Diarrhoeal Disease Research, Bangladesh), Dhaka, Dhaka, Bangladesh; Institute of Epidemiology, Disease Control and Research (IEDCR), Dhaka, Dhaka, Bangladesh; Institute of Epidemiology, Disease Control & Research (IEDCR), Dhaka, Dhaka, Bangladesh; Institute of Epidemiology, Disease Control & Research (IEDCR), Dhaka, Dhaka, Bangladesh; icddr,b (International Centre for Diarrhoeal Disease Research, Bangladesh), Dhaka, Dhaka, Bangladesh; Institute of Epidemiology, Disease Control & Research (IEDCR), Dhaka, Dhaka, Bangladesh; Institute of Epidemiology, Disease Control & Research (IEDCR), Dhaka, Dhaka, Bangladesh; Institute of Epidemiology, Disease Control & Research (IEDCR), Dhaka, Dhaka, Bangladesh; Institute of Epidemiology, Disease Control & Research (IEDCR), Dhaka, Dhaka, Bangladesh; Institute of Epidemiology, Disease Control & Research (IEDCR), Dhaka, Dhaka, Bangladesh; icddr,b (International Centre for Diarrhoeal Disease Research, Bangladesh), Dhaka, Dhaka, Bangladesh; icddr,b (International Centre for Diarrhoeal Disease Research, Bangladesh), Dhaka, Dhaka, Bangladesh; icddr,b (International Centre for Diarrhoeal Disease Research, Bangladesh), Dhaka, Dhaka, Bangladesh; Institute of Epidemiology, Disease Control & Research (IEDCR), Dhaka, Dhaka, Bangladesh; icddr,b (International Centre for Diarrhoeal Disease Research, Bangladesh), Dhaka, Dhaka, Bangladesh; Institute of Epidemiology, Disease Control & Research (IEDCR), Dhaka, Dhaka, Bangladesh; Institute of Epidemiology, Disease Control & Research (IEDCR), Dhaka, Dhaka, Bangladesh; Institute of Epidemiology, Disease Control & Research (IEDCR), Dhaka, Dhaka, Bangladesh; Institute of Epidemiology, Disease Control & Research (IEDCR), Dhaka, Dhaka, Bangladesh; Institute of Epidemiology, Disease Control & Research (IEDCR), Dhaka, Dhaka, Bangladesh; Institute of Epidemiology, Disease Control & Research (IEDCR), Dhaka, Dhaka, Bangladesh; Institute of Epidemiology, Disease Control & Research (IEDCR), Dhaka, Dhaka, Bangladesh; Institute of Epidemiology, Disease Control & Research (IEDCR), Dhaka, Dhaka, Bangladesh; icddr,b (International Centre for Diarrhoeal Disease Research, Bangladesh), Dhaka, Dhaka, Bangladesh; icddr,b (International Centre for Diarrhoeal Disease Research, Bangladesh), Dhaka, Dhaka, Bangladesh; icddr,b (International Centre for Diarrhoeal Disease Research, Bangladesh), Dhaka, Dhaka, Bangladesh; icddr,b (International Centre for Diarrhoeal Disease Research, Bangladesh), Dhaka, Dhaka, Bangladesh; icddr,b (International Centre for Diarrhoeal Disease Research, Bangladesh), Dhaka, Dhaka, Bangladesh; icddr,b (International Centre for Diarrhoeal Disease Research, Bangladesh), Dhaka, Dhaka, Bangladesh; Institute of Epidemiology, Disease Control & Research (IEDCR), Dhaka, Dhaka, Bangladesh; Institute of Epidemiology, Disease Control & Research (IEDCR), Dhaka, Dhaka, Bangladesh; Institute of Epidemiology, Disease Control & Research (IEDCR), Dhaka, Dhaka, Bangladesh; icddr,b (International Centre for Diarrhoeal Disease Research, Bangladesh), Dhaka, Dhaka, Bangladesh; US CDC, Atlanta, Georgia; Centers for Disease Control and Prevention (CDC), Atlanta, Georgia; icddr,b (International Centre for Diarrhoeal Disease Research, Bangladesh), Dhaka, Dhaka, Bangladesh; icddr,b (International Centre for Diarrhoeal Disease Research, Bangladesh), Dhaka, Dhaka, Bangladesh; United States Department of Agriculture (USDA), Manhattan, Kansas; icddr,b (International Centre for Diarrhoeal Disease Research, Bangladesh), Dhaka, Dhaka, Bangladesh; Centers for Disease Control and Prevention (CDC), Atlanta, Georgia; Institute of Epidemiology, Disease Control and Research (IEDCR), Dhaka, Dhaka, Bangladesh

## Abstract

**Background:**

Nipah virus (NiV), a high-risk pathogen with pandemic potential, causes near-annual outbreaks in Bangladesh. Since January 2025, the Institute of Epidemiology, Disease Control and Research (IEDCR) and icddr,b have investigated three NiV outbreaks.

**Table 1:**
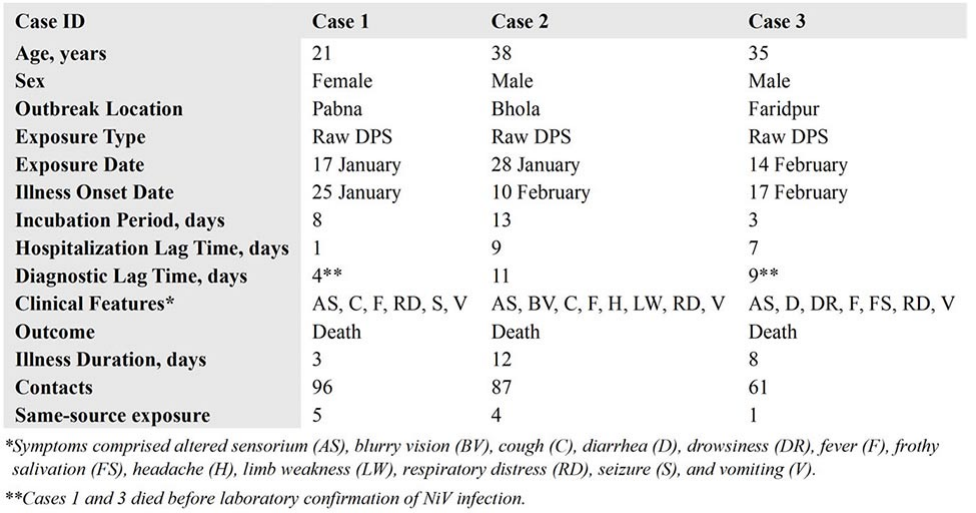
Demographic, Epidemiological, and Clinical Findings of Nipah Cases in the 2025 Outbreaks in Bangladesh

**Table 2:**
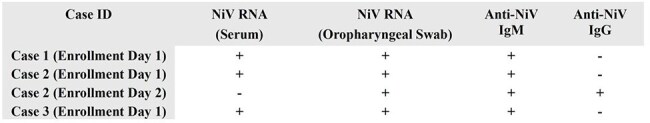
Laboratory Test Results of Nipah Cases in the 2025 Outbreaks in Bangladesh

**Methods:**

Structured and informal interviews with case families, contacts, and communities explored exposures, disease history, risk behaviors, possible spillover, and transmission routes. Oropharyngeal swabs and sera were tested in high-risk contacts and same-source exposed individuals for NiV RNA (RT-qPCR) and anti-NiV IgM (ELISA).

**Figure 1:**
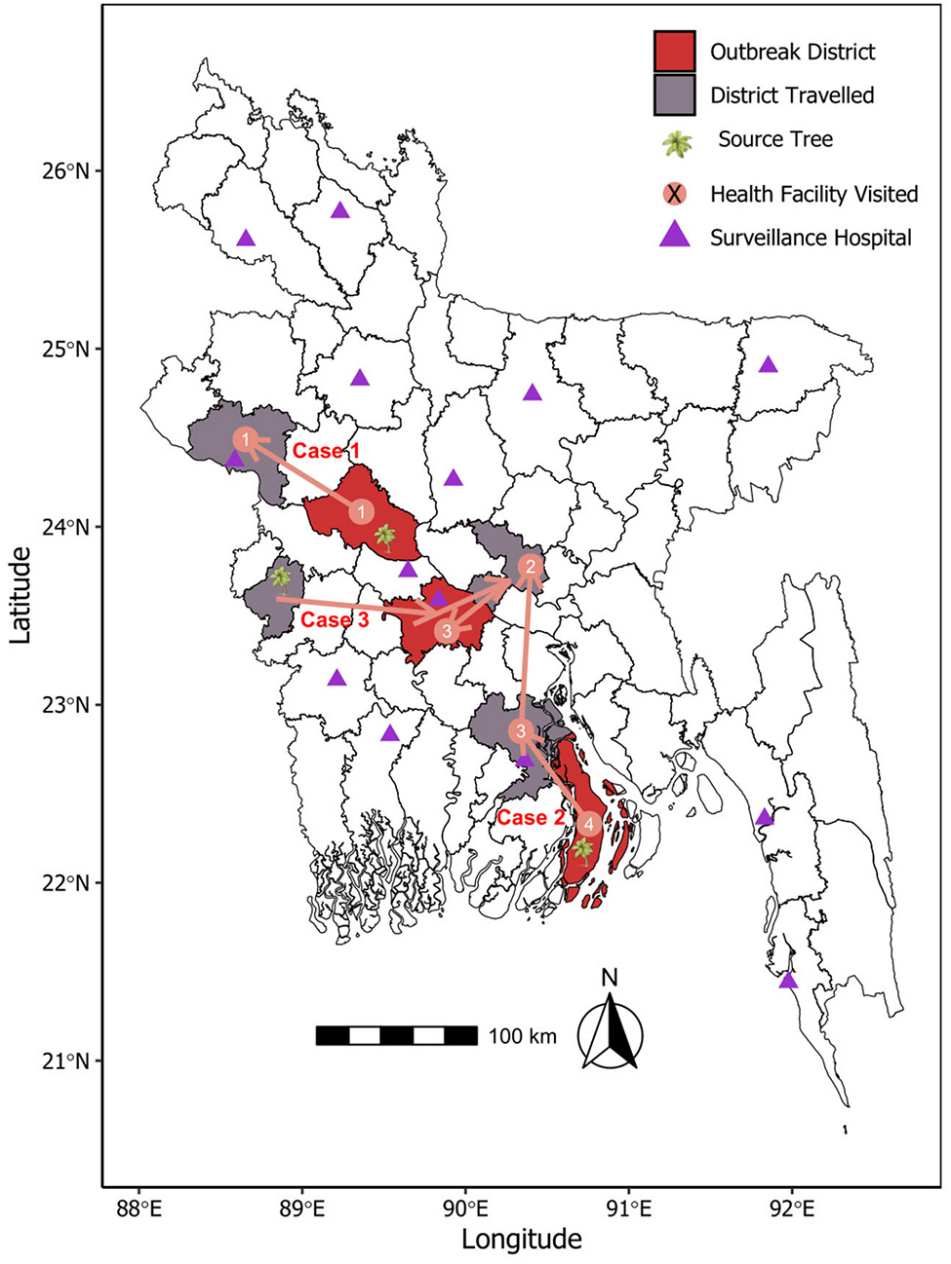
Geospatial Mapping of Key Locations of the 2025 Nipah Outbreaks in Bangladesh

**Results:**

Three sporadic NiV outbreaks were detected in urban or peri-urban areas of Pabna, Bhola (first-ever case), and Faridpur districts between January 29 and February 26 in 2025. All were primary cases linked to raw date palm sap (DPS) consumption. Bat roosts near source date palm trees (2-13 km) housed 20-500 *Pteropus mediu*s bats each. Cases tested NiV-positive within 4-11 days of symptom onset (incubation period: 3-13 days) and 2-3 days post-hospitalization (hospitalization lag time: 1-9 days). All presented with fever, varied neurological symptoms (seizure, blurry vision, altered sensorium, limb weakness), and respiratory distress, and died within 3-12 days of onset. We identified 244 contacts; 10 same-source exposures (raw DPS), of which 7 were also contacts. All 140 high-risk contacts and same-source exposed individuals tested NiV-negative. Investigations revealed widespread raw DPS consumption, even among the urban populations and beyond past outbreak areas, daytime harvesting for sweetness instead of overnight collection practice, and nighttime consumption in contrast to traditional morning intake.

**Conclusion:**

As in 2024, fatality in all 2025 NiV cases confirms sustained NiV virulence in Bangladesh. Widespread raw DPS consumption and delays in care-seeking highlight the critical need for community awareness. To map new exposure risks and comprehend community risk perception, further investigation is needed into evolving DPS collection and consumption practices. Surveillance at lower-tier facilities, developing local diagnostic capacity, and physician training could aid early detection, reduce diagnostic delays, and improve healthcare delivery and patient outcomes.

**Disclosures:**

All Authors: No reported disclosures

